# Descriptors of Cytochrome Inhibitors and Useful Machine Learning Based Methods for the Design of Safer Drugs

**DOI:** 10.3390/ph14050472

**Published:** 2021-05-17

**Authors:** Tyler C. Beck, Kyle R. Beck, Jordan Morningstar, Menny M. Benjamin, Russell A. Norris

**Affiliations:** 1 Drug Discovery & Biomedical Sciences, Medical University of South Carolina, 280 Calhoun Street, QF204, Charleston, SC 29424-2303, USA; beckt@musc.edu (T.C.B.); benjamim@musc.edu (M.M.B.); 2 Regenerative Medicine & Cell Biology, Medical University of South Carolina, 171 Ashley Avenue, 604F CRI, Charleston, SC 29424-2303, USA; morningj@musc.edu; 3 College of Pharmacy, The Ohio State University, 217 Lloyd M Parks Hall, 500 West 12th Ave., Columbus, OH 43210, USA; beck.735@buckeyemail.osu.edu

**Keywords:** CYP3A4, CYP2D6, CYP2C19, CYP2C9, CYP1A2, cheminformatics, cytochrome

## Abstract

Roughly 2.8% of annual hospitalizations are a result of adverse drug interactions in the United States, representing more than 245,000 hospitalizations. Drug–drug interactions commonly arise from major cytochrome P450 (CYP) inhibition. Various approaches are routinely employed in order to reduce the incidence of adverse interactions, such as altering drug dosing schemes and/or minimizing the number of drugs prescribed; however, often, a reduction in the number of medications cannot be achieved without impacting therapeutic outcomes. Nearly 80% of drugs fail in development due to pharmacokinetic issues, outlining the importance of examining cytochrome interactions during preclinical drug design. In this review, we examined the physiochemical and structural properties of small molecule inhibitors of CYPs 3A4, 2D6, 2C19, 2C9, and 1A2. Although CYP inhibitors tend to have distinct physiochemical properties and structural features, these descriptors alone are insufficient to predict major cytochrome inhibition probability and affinity. Machine learning based in silico approaches may be employed as a more robust and accurate way of predicting CYP inhibition. These various approaches are highlighted in the review.

## 1. Introduction

Roughly seventy-four percent (74%) of all physician office visits involve drug therapy, with 2.9 billion drugs prescribed annually [1]. These prescriptions accounted for USD 335 billion in patient costs in 2020 [2]. Half of Americans take at least one prescription drug and nearly a quarter of persons use three or more medications concomitantly [1]. An estimated 6.7% of hospitalized patients experience an adverse drug reaction with a fatality rate of 0.32%, responsible for 106,000 deaths annually [3]. These data suggest adverse drug reactions are the fourth leading cause of death in the United States [3,4,5]. Furthermore, these statistics exclude ambulatory adverse drug reactions and are therefore likely an underrepresentation of the morbidity and mortality burden of adverse drug reactions. Nearly 350,000 adverse drug reactions occur in nursing homes annually, where polypharmacy is commonplace [6]. Many of these drug–drug interactions are avoidable with proper patient medication management and by deprescribing. While efforts to reduce drug–drug interactions are important, a reduction in the number of medications cannot always be achieved without impacting therapeutic outcomes.

**Figure 1:**
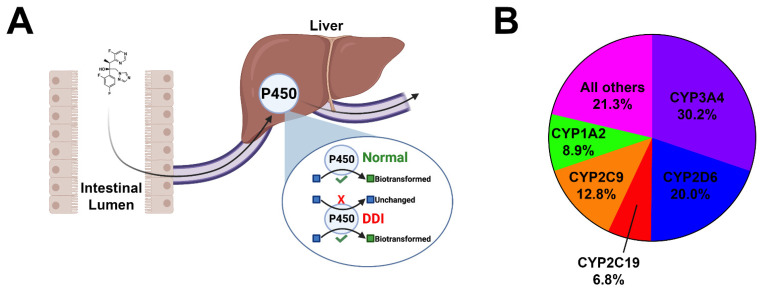
Cartoon depiction of cytochrome inhibition and pie chart. (**A**) Orally bioavailable drugs are absorbed from the intestinal lumen into the mesenteric capillaries and transported to the liver via the portal vein. Many drugs undergo phase I biotransformation by major cytochromes within hepatocytes. This process enzymatically converts lipid-soluble compounds to more water-soluble compounds to facilitate the excretion. In the event of polypharmacy and/or the co-administration of CYP inhibitors, serum accumulation of unmetabolized drugs may occur, leading to untoward toxicities. (**B**) Pie chart demonstrating the breakdown of the five major cytochromes and their contributions to the oxidative metabolism of xenobiotics and organic endogenous molecules.

Adverse drug reactions commonly occur due to drug–drug interactions as a result of major cytochrome P450 (CYP) inhibition [7]. CYPs are heme-containing membrane-bound enzymes that predominantly reside in the smooth endoplasmic reticulum and mitochondria of hepatocytes and in the intestines [8,9]. In mammals, 57 CYP isoforms have been identified with the function of performing the oxidative metabolism of xenobiotics and endogenous compounds [8]. Of the 57 CYP isoforms, 5 CYPs (CYPs 3A4, 2D6, 2C19, 2C9, and 1A2) are responsible for the metabolism of more than 80% of clinically used drugs [7] (Figure 1). Enzyme inhibition occurs when two co-administered drugs share an identical mechanism of biotransformation and compete for metabolism in the same enzyme receptor site [9]. As such, the more potent inhibitor will prevail, interrupting biotranformation, resulting in the diminished metabolism of the competing drug. This results in elevated serum drug levels of the unmetabolized drug, increasing the probability of adverse toxicological outcomes. This is especially important for drugs with a narrow therapeutic index, such as chemotherapeutics. It is important to note that inhibitors of major cytochromes can promote adverse drug reactions when administered as monotherapy. Inhibitors of major cytochromes may alter their own metabolism at high doses, leading to toxic plasma drug accumulation. These interactions pose a significant safety risk and should be examined closer during the initial drug design process. The CYP system and drug biotransformation has been reviewed extensively elsewhere [10].

Traditionally, pharmacokinetics has been neglected in preclinical drug design. Roughly four in five investigational new drugs fail as a result of absorption, distribution, metabolism, excretion and toxicity (ADMET) issues [11]. Tools that assist in ADMET prediction may be advantageous in early drug design by reducing the drug attrition rate in advanced preclinical- and early clinical-stage studies. In particular, the design of drugs devoid of major cytochrome inhibition would reduce the burden of drug–drug interactions in patients where polypharmacy is unavoidable. Several reviews exist that focus on platforms that utilize a diverse range of prediction methods [12,13,14,15,16,17,18,19,20]. Many of these reviews focus on outdated tools, as well as platforms that are exclusively available through a fee-based licensing model. More recently, numerous machine learning-based platforms have emerged that are user-friendly, are not as computationally expensive as pre-existing methods, and demonstrate improved prediction accuracy. In this review, we will explore the various physiochemical and structural descriptors of CYP inhibitors, as well as superior machine learning prediction platforms freely available to investigators.

## 2. Physiochemical Properties

**Figure 2:**
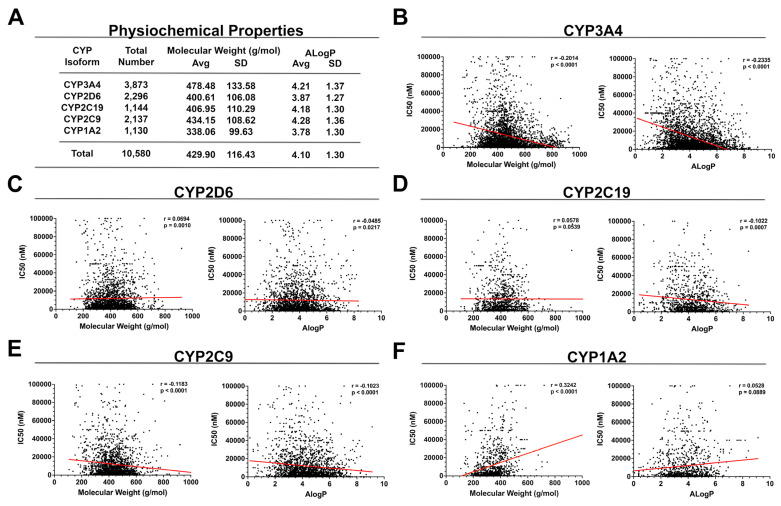
Summary of the physiochemical properties of major cytochrome inhibitors and their impact on half-maximal inhibitor concentration (IC_50_). (**A**) Molecular weight and lipophilicity of validated inhibitors of each individual cytochrome. (**B**–**F**) Graphs representing IC_50_ versus either molecular or ALogP for CYPs 3A4 (**B**), 2D6 (**C**), 2C19 (**D**), 2C9 (**E**), and 1A2 (**F**). CYP3A4 and CYP2C9 inhibitors demonstrated a weak statistically significant negative relationship between molecule size, lipophilicity, and IC_50_ (i.e., larger, more lipophilic molecules, tend to have lower IC_50_ values); however, the exact opposite trend was observed for CYP1A2. No relationship was identified for CYPs 2D6 and 2C19.

Historically, physiochemical properties have been used as key descriptors to predict CYP inhibition liability. Cytochrome P450 enzymes are inherently lipophilic and thus have a predilection for lipophilic substrates [21,22,23,24,25]. It has been hypothesized that molecular weight and lipophilicity represent key physiochemical characteristics useful in predicting CYP binding affinity [21,26,27,28]. In further exploring this hypothesis, we examined CYP inhibitors deposited in the ChEMBL database to further illustrate the relationship between molecular weight, lipophilicity, and the IC_50_ of various major cytochromes (Figure 2). ChEMBL hosts a database of over one million small molecules with reported bioactivities and calculated physiochemical properties [29]. Data on the inhibitors of each individual cytochrome were extracted for further analysis. Duplicates, as well as data points that were flagged as potentially unreliable by ChEMBL, were withheld from the analysis. The inhibitors of major cytochromes tend to be lipophilic in nature (Figure 2A). Inhibitors of CYP3A4, CYP2C19, and CYP2C9 were especially lipophilic (ALogP = 4.21, 4.18, and 4.28, respectively). Inhibitors of CYP3A4 and CYP2C9 tend to be larger structures, whereas inhibitors of CYP1A2 tend to be small. These data make sense considering the shape and size of the CYP 3A4, 2C9, and 1A2 enzyme receptor sites, which will be reviewed in Section 3. It should be noted, however, that although inhibitors of CYP3A4 and CYP2D6 tend to be larger in size, many of their substrates are small-sized molecules, such as furanocoumarin, diltiazem, mifepristone, buproprion, and fluoxetine.

We then examined the relationship between molecular weight, lipophilicity (ALogP) and IC_50_ by performing simple linear regression and Pearson’s correlation analyses. In doing so, we identified several statistically significant correlations between physiochemical properties and CYP half-maximal inhibitory concentration; however, these mild correlations do not adequately explain the majority of the data (Figure 2B–F). Next, we performed a multiple linear regression analysis for each major cytochrome to further assess how multiple explanatory variables, such as molecular weight and lipophilicity, impact the half-maximum inhibitory concentration (Supplemental Materials Tables S1–S5). A statistically significant relationship (*p* < 0.05) between molecular weight, lipophilicity, and IC_50_ was identified for CYPs 3A4 (R = 0.136), 2C19 (0.125), 2C9 (0.132), and 1A2(0.309). A statistically significant relationship was not identified for CYP2D6 (R = 0.024, *p* = 0.510). As observed with the simple linear regression data, exclusively weak relationships were observed that poorly describe the data. Although CYP inhibitors tend to fit a distinct physiochemical profile, these descriptors alone are insufficient to predict the probability of cytochrome inhibition and CYP half-maximal inhibitory concentration.

## 3. Structural Properties

The individual CYP isoforms come in different shapes, sizes, and compositions, contributing to unique substrate specificity profiles. The key structural features of a few important CYP inhibitors have been elucidated using X-ray crystallography, pharmacophore modeling, and various other in silico approaches [30,31,32,33,34,35,36,37,38]. In particular, the structural features of CYP3A4 inhibitors have been well studied [30,39,40,41,42,43]; however, data on the specific structural features of inhibitors for the other four major cytochromes are limited and warrant further investigation. In this section, we review the structural features of CYP enzymes and highlight the structural features of well-known, potent CYP inhibitors. In particular, emphasis is placed on molecular weight, surface area, lipophilicity (LogP), rotatable bonds, hydrogen bond acceptors, and hydrogen bond donors. The molecular weights and surface areas of small molecules are key driving factors that dictate the strength of binding interactions. In general, an enzyme or protein with a small binding cavity will be more compatible with a smaller drug, etc. Lipophilicity is an important descriptor that is used to predict CYP interactions. A majority of CYP inhibitors possess numerous aromatic rings and hydrophobic moieties capable of interacting with lipophilic CYP-active sites. Rotatable bonds determine the flexibility of a drug molecule and are important for inhibitor binding interactions with certain CYPs (i.e., CYP3A4), and less important for other CYPs (i.e., CYPs 2D6 and 1A2). Lastly, hydrogen bond acceptor and donor moieties form specific binding interactions with active site amino acids. These interactions were further explored using molecular docking, which was performed using previously described methods [44,45,46].

### 3.1. CYP3A4

**Figure 3:**
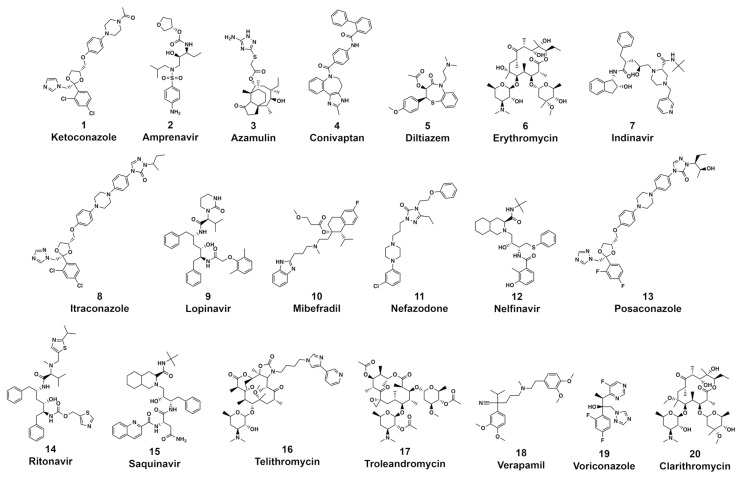
Structures of the 20 most commonly prescribed high-affinity CYP3A4 inhibitors.

**Figure 4:**
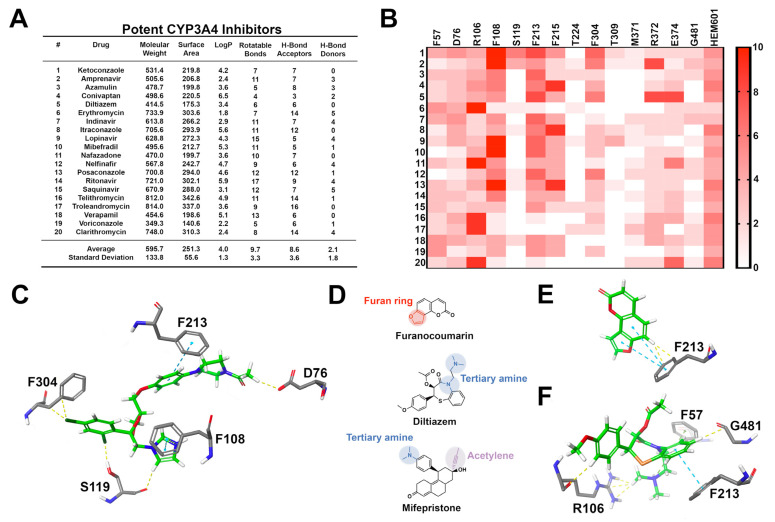
Structural investigation of the 20 most commonly prescribed CYP3A4 inhibitors. (**A**) CYP3A4 inhibitors are large, lipophilic, and flexibility molecules capable of accepting numerous hydrogen bonds. Molecular weight is represented as g/mol or Daltons (Da) and surface area is represented in angstroms (Å). Values were calculated using pkCSM ADMET [38]. (**B**) Heat map representing the frequency of ligand-to-amino acid binding interactions in the top ten most energetically favorable binding poses in docking against CYP3A4 (PDB ID: 1W0E). (**C**) Docking image demonstrating ketoconazole, a potent CYP3A4 inhibitor, making numerous pi–pi stacking interactions (blue dotted lines) with F108, F213, and F304, as well as energetically favorable contacts (yellow dotted lines) with D76 and S119. (**D**) Moieties commonly associated with high-affinity CYP inhibition in the literature, including the presence of a furan ring, tertiary amine, or acetylene group [39]. (**E**) 3D docking image of furanocoumarin, a CYP3A4 inhibitor found in the skin of grapefruit. Furanocoumarin makes several pi–pi stacking interactions and energetically favorable contacts. The furan ring of furanocoumarin specifically lends to pi–pi stacking interactions within the CYP3A4 receptor site. (**F**) 3D docking image of diltiazem, an antihypertensive drug and calcium channel blocker known for inhibiting CYP3A4. Diltiazem forms many pi–pi stacking interactions and energetically favorable contacts.

CYP3A4 is the most widely expressed and clinically significant cytochrome responsible for the biotransformation of 30.2% of xenobiotics that undergo hepatic clearance [47]. CYP3A4 is capable of oxidizing a wide array of bulky substrates with many different structural features. The versatile functionality of CYP3A4 is due to its large substrate-binding cavity [47,48]. The active site cavity of CYP3A4 is open and flexible, capable of accommodating volumes ranging from 1173 to 2862 cubic angstroms (Å^3^) [39]. The CYP3A4–ligand complexes formed upon substrate binding are incredibly flexible, explaining the observed ligand promiscuity and diverse range of substrates capable of high-affinity binding interactions [49]. In further examining the 20 most frequently prescribed CYP3A4 inhibitors, we identified shared features in comparing the compounds (Figure 3). The most frequently prescribed high-affinity CYP3A4 inhibitors have a very high molecular weight (595.7 g/mol) and a large surface area (251.3 Å) (Figure 4). Additionally, these structures are highly lipophilic (LogP = 4.0) and have many rotatable bonds and hydrogen bond acceptors (9.7 and 8.6, respectively). These hydrogen bond acceptors are available to interact with key amino acids within the CYP3A4 active site, including arginine 106 (R106), serine 119 (S119), threonine 309 (T309), and arginine 372 (R372). These data are consistent with previous reports, suggesting that potent inhibitors of CYP3A4 are large, lipophilic molecules with a significant degree of flexibility. It is important to note that while CYP3A4 inhibitors are capable of diverse binding poses, there are a few high-energy binding interactions that occur among all high-affinity binders. Molecules with a tertiary amine or imidazole nitrogen frequently coordinate with heme-601 (HEM601) [12,50]. High-affinity CYP3A4 inhibitors are lipophilic, containing one or more aromatic rings capable of forming numerous pi–pi stacking interactions with active phenylalanine (F) residues, including F108, F213, F215, F304, and occasionally F57. A majority of high-affinity inhibitors make polar interactions with D76, R106, S119, and R372. Additionally, CYP3A4 inhibitors typically possess a nitrogen or allylic position as a site of oxidation [48]. Lastly, high-affinity CYP3A4 inhibitors have approximately two hydrogen bond acceptors. These moieties commonly accept hydrogens from aspartate 76 (D76) and E374. Due to the highly flexible nature of the enzyme-binding pocket and the diversity of ligand-binding interactions, predicting drug–drug interactions involving CYP3A4 is difficult. 

In addition to hepatic metabolism, CYP3A4 drug metabolism also occurs in the gastrointestinal (GI) tract. This is true for the oral chemotherapeutic ibrutinib, used to treat chronic lymphocytic leukemia (CLL), which makes roughly equal contributions to hepatic and intestinal CYP3A4 metabolism [51]. Moreover, some agents exclusively inhibit CYP3A4 in the GI tract (minimally effective hepatic CYP3A4), resulting in increased drug concentrations. One specific example is the co-administration of grapefruit juice with drugs that are metabolized by CYP3A4. Grapefruit juice irreversibly inhibits intestinal, but not hepatic, CYP3A4, increasing systemic drug exposure [52]. These examples highlight the significance of CYP3A4 inhibition in tissues outside of the liver. 

### 3.2. CYP2D6

**Figure 5:**
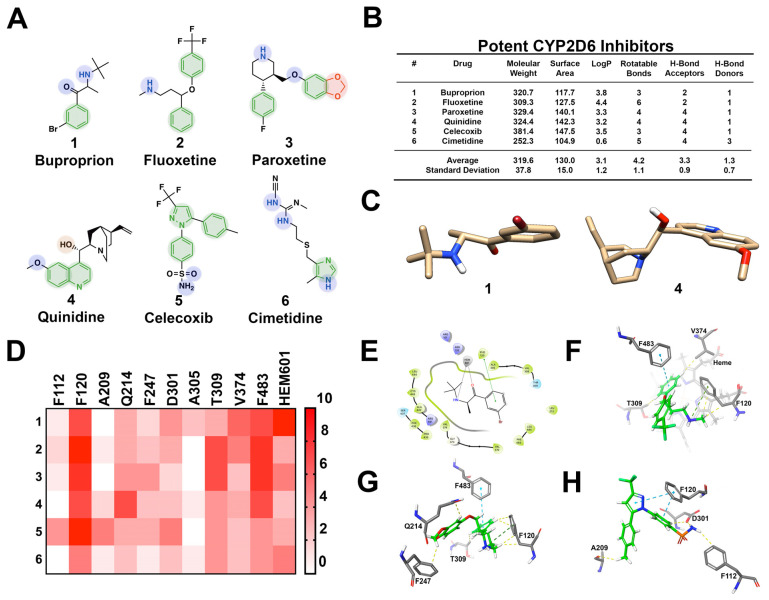
Structural investigation of 6 well-known high-affinity CYP2D6 inhibitors. (**A**) CYP2D6 inhibitors commonly have one or multiple aromatic moieties, heterocycles, and primary or secondary amines. Red and green moieties interact with F112, F120, F247, F483, and/or heme-601; blue moieties interact with DD301 and/or heme-601, and orange interact with D301. (**B**) CYP2D6 inhibitors are rigid, moderately sized structures with varying physiochemical properties. (**C**) CYP2D6 inhibitors are flat, planar structures capable of becoming positively charged. (**D**) Heat map representing the frequency of ligand-to-amino acid binding interactions in the top ten most energetically favorable binding poses in docking against CYP2D6 (PDB ID: 3QM4). Key amino acids include F120, Q214, D301, T309, V374, and F483. Frequent interactions are observed with heme as well. (**E**) Two-dimensional ligand interaction map showing the secondary amine and carbonyl group of buproprion coordinating with Heme-601. (**F**) Three-dimensional docking image depicting fluoxetine interaction with F120, T309, V374, and F483. (**G**) Three-dimensional docking image showing paroxetine forming pi–pi stacking interactions with F120, F247, and F83, as well as energetically favorable contacts with Q214 and T309. (**H**) Three-dimensional docking image showing celecoxib interacting with F120, F112, A209, and D301.

CYP2D6 comprises 2% of hepatocyte CYP content and is responsible for the biotransformation of 20% of drugs that undergo hepatic metabolism [53]. The active site cavity of CYP2D6 has a more restricted volume of 510 cubic angstroms (Å^3^) [39,54]. The active site of CYP2D6 is acidic in nature and contains several phenylalanine residues capable of pi–pi stacking with substrates [55]. As such, it has been reported that CYP2D6 has a predilection for basic substrates [56]. In examining six well-known high-affinity CYP2D6 inhibitors, the structural similarities include a primary or secondary amine, heterocycles, and numerous aromatic moieties (Figure 5). These CYP2D6 inhibitors are relatively small in composition and are moderately lipophilic. It should be noted that in examining 2296 CYP2D6 inhibitors, the average size was 400.61 Da, in comparison to 319.6 Da observed in the six most frequently prescribed drugs. Regardless of the discrepancy, the typical CYP2D6 inhibitor is smaller than the average FDA-approved drug (480 Da) [57]. Additionally, CYP2D6 inhibitors are typically flat, planar structures capable of procuring a positive charge. The active site contains two carboxylic acid residues from Q216 and D301 that are critical for substrate recognition and binding selectivity [12]. These acidic residues frequently interact with the primary and/or secondary amine groups present on CYP2D6 inhibitors. The aromatic moieties make energetically favorable pi–pi stacking interactions with F120, F483, and less commonly F112. Potent inhibitors commonly contain heterocycles, primary amines, and carbonyl groups that coordinate with the iron of heme-601 (HEM601) within the CYP2D6-active site. Additionally, the average inhibitor contained 4.2 rotatable bonds and 3.3 hydrogen bond acceptors, most frequently interacting with glutamine 214 (Q214) and T309. CYP2D6 inhibitors contain approximately one hydrogen bond donor, which occasionally interacts with aspartate 301 (D301). Lastly, valine 374 (V374) forms van der Waals interactions with hydrophobic residues of inhibitors.

As described with CYP3A4, it is difficult to predict half-maximal inhibition concentration using physiochemical and structural features alone given the diverse binding capabilities of CYP2D6. A study by Vandenbrink et al. demonstrated that CYP2D6’s inhibition potency is substrate-dependent [58]. In examining the binding kinetics of 20 known inhibitors of CYP2D6 in the presence of four clinically relevant substrates, atypical inhibition kinetics were observed for each individual inhibitor–substrate pairing. The substrates with constrained amine moieties impaired inhibitor binding affinity by more than 5-fold when compared to other structurally unrelated substrates. These data add an additional layer of difficulty in making reliable predictions for cytochromes such as CYP2D6 that interact with a wide range of structurally diverse substrates. Lastly, CYP2D6 is highly polymorphic, with the largest phenotypic variation among the human CYPs [59]. Approximately 80 different allelic variants and 130 genetic variations have been characterized in patients, with phenotypic consequences ranging from loss of function to ultra-rapid metabolization. Therefore, it should be acknowledged that while CYP2D6 predictions may be accurate in a majority of cases, the data will be subject to significant variability due to the genetic heterogeneity present in the global population, which will be an unavoidable roadblock in preclinical drug design.

### 3.3. CYP2C19

**Figure 6:**
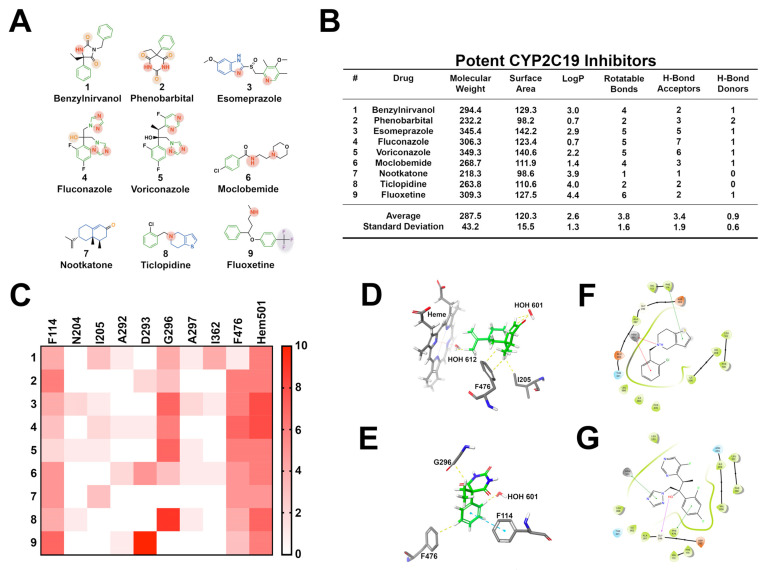
Structural investigation of 9 well-known high-affinity CYP2C19 inhibitors. (**A**) Common structural features seen in CYP2C19 inhibitors include aromatic moieties, heterocycles, carbonyl groups, and aromatic nitrogens. Green moieties interact with F114, F476, and/or heme; blue and red moieties interact with heme; orange interact with G296, and purple interact with water and I205. (**B**) The nine most frequently prescribed CYP2C19 inhibitors are small; however, in examining 1144 CYP219 inhibitors, the average molecule is moderately sized. (**C**) Heat map representing the frequency of ligand-to-amino acid binding interactions in the top ten most energetically favorable binding poses in docking against CYP2C19 (PDB ID: 4GQS). Key amino acids include F114, G296, F476. Frequent interactions are observed with heme-501 as well. (**D**) Three-dimensional ligand docking showing nootkatone interacting with I205, F746 and water molecules within the active site. (**E**) Three-dimensional docking image depicting phenobarbital interacting with F114, G296, F476, and water molecules within the active site. (**F**) Two-dimensional ligand interaction diagram showing the cyclic nitrogen and arylene group of ticlopidine coordinating with heme-501. (**G**) Two-dimensional interaction map showing voriconazole coordinating with heme, while simultaneously interacting with G296 and F476.

CYP2C19 metabolizes 6.8% of drugs that undergo hepatic biotransformation. Despite being considered a minor hepatic cytochrome based on its relative contribution, CYP2C19 is responsible for metabolizing several clinically important drugs, including omeprazole and phenytoin [60]. As such, many pharmacologists view CYP2C19 as a major cytochrome. Inhibitors of CYP2C19 share frequently observed structural features (Figure 6). Structures contain numerous aromatic moieties, heterocycles, carbonyl groups, and aromatic nitrogen atoms. Several well-characterized high-affinity CYP2C19 inhibitors are small-sized molecules, but they tend to be less lipophilic than inhibitors of other cytochromes. In general, however, CYP2C19 inhibitors tend to be medium-sized molecules with variable lipophilicity. These results are consistent with previous reports, claiming that lipophilicity does not impact CYP2C19 inhibitory potency [12,61]. On average, these molecules are less flexible than typical CYP inhibitors (3.8 rotatable bonds) and have approximately three hydrogen bond acceptors. The hydrogen bond acceptor moieties commonly interact with asparagine 204 (N204). It is important to note that the location of the hydrogen bond acceptors helps dictate binding affinity. CYP2C19 inhibitors should have a minimum of two hydrogen bond acceptors within close proximity to a site of oxidation [12]. CYP2C19 inhibitors contain heterocycles and heterocyclic nitrogen atoms that frequently coordinate with Heme-501 (HEM501). Numerous pi–pi stacking interactions occur between small-molecule arylene groups and F114 and F476. Additionally, G296 forms frequent interactions with hydroxyl and carbonyl groups present on CYP2C19 inhibitors. CYP2C19 inhibitors make numerous hydrophobic contacts with isoleucine 205 (I205), alanine 292 (A292), glycine 296 (G296), A297, and I362.

Similar to CYP2D6, a variety of genetic polymorphisms exist within the CYP2C19 isoform. Roughly 5% of Caucasians and 20% of Asian populations possess genetic variations resulting in reduced or complete loss of enzyme function [61,62,63,64]. Although less common, genetic variations may also result in enhanced metabolic activity, which also impacts drug handling and excretion. These polymorphisms complicate predictions at the population level and should be taken into consideration.

### 3.4. CYP2C9

**Figure 7:**
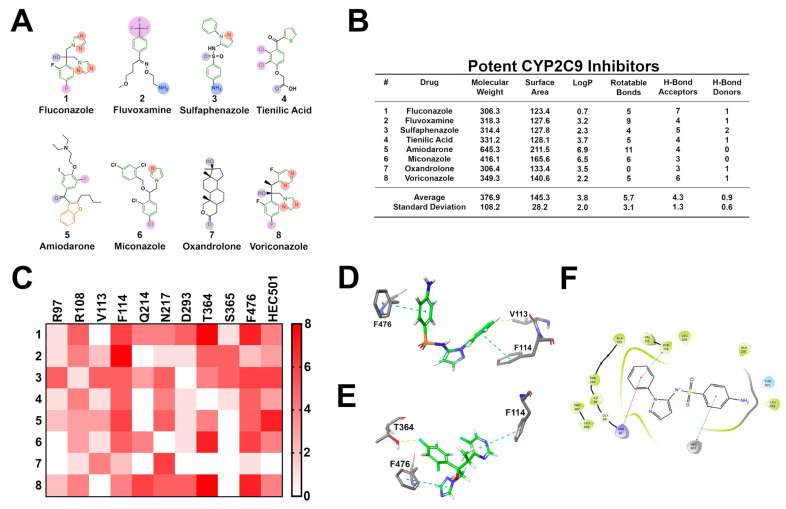
Structural investigation of 8 well-known high-affinity CYP2C9 inhibitors. (**A**) The common structural features seen in CYP2C9 inhibitors include aromatic rings, aromatic nitrogens, heterocycles, primary amines, and halogens. Purple moieties interact with N217; magenta interact with T364; green interact with F114, F476, and/or heme; blue interact with S365; red interact with Q214; and orange interact with heme. (**B**) Molecules tend to be moderately sized with varying lipophicity. CYP2C9 inhibitors are moderately flexible and possess numerous H-bond acceptors. (**C**) Heat map representing the frequency of ligand-to-amino acid binding interactions in the top ten most energetically favorable binding poses in docking against CYP2C9 (PDB ID: 1OG5). Ligand interactions are highly diverse, with key interactions including R108, F114, F476, and heme-501. (**D**) Three-dimensional docking image depicting sulfaphenazole forming pi–pi stacking interactions between F114 and F476, as well as energetically favorable contacts with V113. (**E**) Three-dimensional docking image portraying voriconazole interacting with F114, T364, and F476. (**F**) Two-dimensional ligand interaction map depicting sulfaphenazole interacting with R97 and F114, while coordinating with the iron of heme-501.

CYP2C9 is responsible for the biotransformation of 12.8% of drugs that undergo hepatic metabolism. In particular, CYP2C9 plays a key role in the metabolism of commonly used medications, such as non-steroidal anti-inflammatory drugs (NSAIDs) and warfarin [12]. The active site of CYP2C9 is rather large and has a cavity volume ranging from 978 to 1271 cubic angstroms (Å^3^) [39,65,66]. Inhibitors of CYP2C9 are typically aromatic compounds with heterocycles, aromatic nitrogens, primary amines, and halogens (Figure 7). Heterocycles, aromatic nitrogens, and primary amines coordinate to the iron of heme-501 (HEC501). Aromatic moieties form extensive pi–pi stacking interactions with F114 and F476. Halogens commonly interact with T364. Additionally, carbonyl, hydroxyl, and carboxylate groups interact with R108. These particular interactions lock the substrate into a position with close proximity to the iron allowing for oxidative metabolism to occur [67]. CYP2C9 inhibitors perform a large array of binding poses; however, binding with R108, F114, and R476 is most commonly observed in the most energetically favorable docking interactions. CYP2C9 inhibitors tend to be medium-sized semi-flexible molecules that are moderately lipophilic and acidic, with a minimum of two to four hydrogen bond acceptors. These hydrogen bond acceptors most commonly interact with R97, R108, N217, T364, and S365. As seen with the other major cytochromes, CYP2C9 is highly polymorphic, with more than 20 activity-altering genetic variants identified [67,68].

### 3.5. CYP1A2

**Figure 8:**
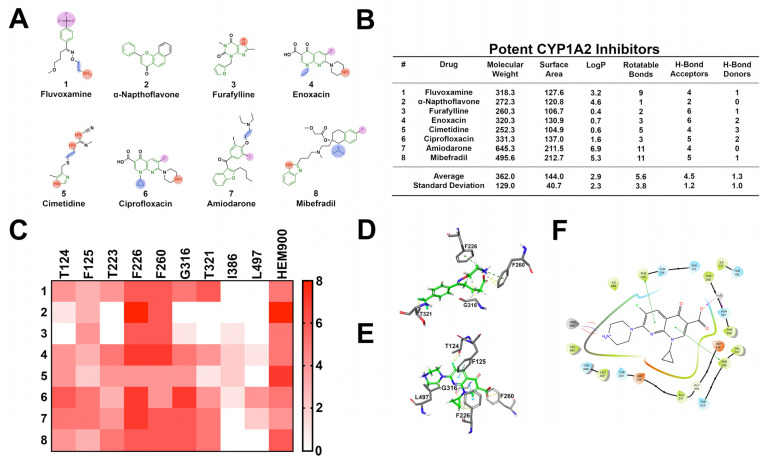
Structural investigation of the 8 most well-characterized high-affinity CYP1A2 inhibitors. (**A**) Common structural features seen in CYP1A2 inhibitors include aromatic rings, heterocycles, primary amines, and halogens. Magenta moieties interact with T124 or T321; green interact with F125, F226, F260, and/or heme; blue interact with G316; red interact with F226, F260 and/or heme. (**B**) CYP1A2 inhibitors are small, planar structures and are moderately flexible with numerous H-bond acceptors. (**C**) Heat map representing the frequency of ligand-to-amino acid binding interactions in the top ten most energetically favorable binding poses in docking against CYP1A2 (PDB ID: 2HI4). Ligand interactions are highly diverse, with key interactions including T124, T223, F226, F260, G316, T321, and heme-900. (**D**) Three-dimensional docking image depicting Fluvoxamine interacting with F226, F260, G316, and T321. (**E**) Three-dimensional docking image portraying ciprofloxacin interacting with T124, F125, F226, F260, G316, and L497. (**F**) Two-dimensional ligand interaction map depicting ciprofloxacin interacting with F125 and F226, while coordinating with heme-900.

CYP1A2 is responsible for the biotransformation of 8.9% of drugs that undergo hepatic metabolism, including antipsychotics and antibiotics [69]. Additional CYP1A2 substrates include caffeine, paracetamol, and aflatoxin B1. Interestingly, CYP1A2 activity is commonly induced by dietary constituents, including various vegetables and polyaromatic hydrocarbons, whereas ingredients such as cumin and turmeric can inhibit enzyme function [70,71]. The active site of CYP1A2 is small and planar with a volume of 375 to 390 cubic angstroms (Å^3^), and thus CYP1A2 substrates tend to be small, planar molecules [12,39]. In examining eight well-known high-affinity CYP1A2 inhibitors, the shared structural features include multiple aromatic moieties, heterocycles, secondary amines, and halogens (Figure 8). These high-affinity CYP1A2 inhibitors are small, moderately lipophilic, aromatic structures with 5.6 rotatable bonds and 4.5 hydrogen bond acceptors. Molecular docking studies indicate a diverse range of binding interactions. Key interactions include pi–pi stacking with F125, F226, and F260, the coordination of secondary amines and aromatic moieties with heme-900 (HEM900), and halogen interactions with T124 and T321. Amino acids such as T124, T223, and T321 can function as hydrogen donors to inhibitors. Lastly, I386 and L497 form Van der Waals interactions with hydrophobic moieties. 

### 3.6. Summary

Understanding the ADMET properties of compounds early in pre-clinical drug design is paramount to lead selection and clinical success. Although key physicochemical and structural descriptors are shared among CYP inhibitors (Table 1), these features on their own are insufficient for predicting CYP inhibition and binding affinity. The most effective means of predicting CYP inhibition is through the use of computational approaches that incorporate physiochemical and structural descriptors from large data sets, while using advanced algorithms to take into consideration molecule flexibility (number of rotatable bonds), the number of hydrogen bond donors and acceptors, pharmacophores, molecule charge, prospective metabolites, and other key descriptors. Machine learning-based in silico approaches will be reviewed in Section 4.

## 4. Machine Learning-Based Methods

**Figure 9:**
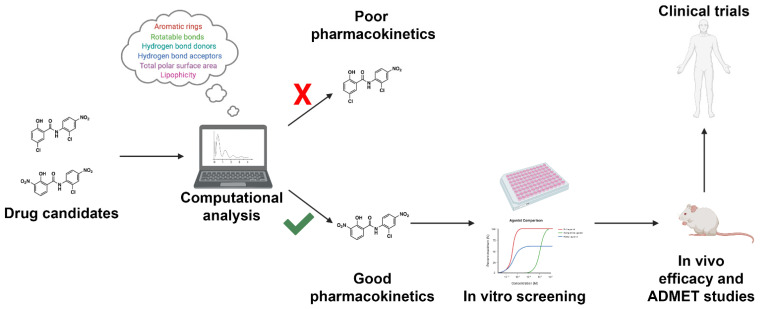
Workflow for incorporating ADMET predictions into early-stage drug design and development. Drug candidates can be readily screened using high-throughput computational approaches. Drugs with acceptable pharmacokinetic parameters as defined by the investigator will proceed to in vitro screening. Drugs with unacceptable pharmacokinetic properties will be triaged early in drug development. The following approach will result in a reduction in ADMET-related drug failures, reducing the cost of drug discovery efforts.

Current in vitro and in vivo experimental approaches to investigate drug ADMET properties are highly effective in assessing pharmacokinetics; however, such approaches are extraordinarily expensive and are commonly performed late in drug development during IND-enabling studies [13]. In silico investigation of CYP interactions early in drug development allows investigators to select for compounds that are less likely to fail in late-stage preclinical or early clinical trials due to undesirable pharmacokinetics (Figure 9). Such tools exist and are effective at predicting CYP inhibitors, common sites of metabolism (SoMs), and prospective drug metabolites. In this section, we highlight computational approaches available for ADMET predictions, focusing primarily on CYP inhibition and metabolism. The aim of this discussion will be to highlight emerging platforms that utilize machine learning-based methods, with a special emphasis placed on applications that are free to the user and available through a public webserver. A complete overview of platforms available through a fee-based licensing model, as well as older free prediction methods and methods for analyzing CYP molecular dynamics, are reviewed elsewhere [13,14,15,16,17,18,19,20]. 

Various platforms capable of making enzyme-inhibitor predictions exist. These techniques use an array of methods, ranging from molecular docking with flexible multi-dimensional QSAR to machine learning based approaches. It should be noted that flexible docking-based methods, such as VirtualToxLab, are computationally expensive, take longer to receive results, and commonly require a software license [13,72]. Machine learning-based tools are capable of producing accurate results in a timely fashion and can be hosted via a publicly available web server. These platforms tend to be more user-friendly, and are commonly able to screen a large number of structures. These platforms each use unique descriptors and utilize their own methods of data generation. As such, the accuracy of said platforms is subject to variability, contingent on the descriptors and molecular fingerprints used, the number of input structures used to build and train the model, and how the data are interpreted. In this section, we review the best publicly available machine learning-based platforms free to the scientific community.

### 4.1. pkCSM

pkCSM is a free, publicly available web service (http://structure.bioc.cam.ac.uk/pkcsm; accessed on 8 February 2021) that offers a wide range of ADMET predictions [50,73]. pkCSM retains no chemical information submitted to the server and is capable of producing results in five minutes or less. Structural information is input to the server as a SMILES code and submitted for analysis. The platform is able to predict the results of key ADMET experiments commonly performed in preclinical drug discovery. Absorption predictions include caco-2 permeability, water solubility, human intestinal absorption, p-glycoprotein substrate or inhibitor, and skin permeability. Distribution predictions include the volume of distribution, the fraction unbound, and blood–brain barrier permeability. Metabolic predictions include cytochrome P450 inhibitors (CYPs 3A4, 2D6, 2C19, 2C9, and 1A2), as well as CYP2D6/3A4 substrates. The excretion predictions made include renal OCT2 substrate and total clearance. Lastly, toxicity predictions include rat LD50, AMES toxicity, T. Pyriformis toxicity, minnow toxicity, maximum tolerated dose, oral rat chronic toxicity, hepatotoxicity, skin sensitization, and hERG I and II inhibitors. pkCSM predictions are made using graph-based signatures to train, test, and predict central ADMET properties for drug development. Exact details on the number of structures used to train each individual parameter are available online (http://biosig.unimelb.edu.au/pkcsm/static/help/pkcsm_theory.pdf; accessed on 8 February 2021). Input molecules are evaluated by the platform based on key descriptors: (1) toxicophore fingerprint, (2) atomic pharmacophore frequency count (hydrophobic, aromatic, hydrogen acceptor, hydrogen donor, positive ionizable, and negative ionizable), and (3) physiochemical and structural properties, including lipophilicity (logP), molecular weight, surface area, number of rotatable bonds, and many more. pkCSM is highly accurate, with Q/AUC values meeting or exceeding those of industry standard ADMET prediction methods (CYP3A4: 0.780/0.847; CYP2D6: 0.853/0.843; CYP2C19: 0.808/0.879; CYP2C9: 0.807/0.868; CYP1A2: 0.802/0.876). Although platforms with comparable or slightly superior CYP inhibitor predictive values exist, pkCSM is capable of making highly accurate predictions on a wide range of relevant ADMET parameters, designating it one the most well-rounded applications available.

### 4.2. DeepCyp

DeepCYP is a free, publicly available web service (http://repharma.pku.edu.cn/deepcyp/home.php; accessed on 8 February 2021) that utilizes a multitask model for concurrent inhibition prediction of the five most clinically relevant CYPs: 3A4, 2D6, 2C19, 2C9, and 1A2 [74]. Test item information is submitted as a mol.2 or comparable file type with results achieved in minutes. Results appear as inhibition probabilities for each individual cytochrome. DeepCYP was built and trained using a multitask autoencoder deep neural network (DNN) with chemical input from the PubChem and BioAssay databases (>13,000 compounds). Descriptors and chemical fingerprints of inhibitors and non-inhibitors were calculated using PaDEL-Descriptor software [74,75]. Totals of 1253 and 688 descriptors for PaDEL-1D&2D and PubChem datasets were used in model training, respectively. Examples of dataset descriptors and fingerprints include atom type electrotopological state (Estate) descriptors, ClogP, molecular linear free energy, element counts, ring counts, simple atom pairs, atom nearest neighbors, and SMARTS patterns. Exact details on model development and training are available online [74]. The multitask DNN DeepCYP platform outperformed single-task models and traditional machine learning methods, with average prediction accuracies of 0.864 for 10-fold cross-validation and 0.887 for the external test datasets. Additionally, the method utilizes linear regression models to determine how each task contributes to predictions, allowing the investigators to identify conditions under which DeepCYP outperforms single task methods. These data may be used to further optimize the model with time. The predictive power of DeepCYP makes it one of the most accurate freely accessible methods available for concurrent CYP inhibition predictions.

### 4.3. SuperCYPsPred

SuperCYPsPred is a free, user-friendly, publicly available web service (http://insilico-cyp.charite.de/SuperCYPsPred/; accessed on 8 February 2021) that uses well-established machine learning methods to predict inhibitor specificity for CYPs 3A4, 2D6, 2C19, 2C9, and 1A2 [7]. The webserver allows for the input of molecules in three different ways: (1) input of a SMILES code, (2) PubChem structure search, or (3) drawing of custom structures in ChemDoodle. The model was developed based on traditional machine learning random forests methods, a commonly employed ensemble learning algorithm that is less prone to class bias and overfitting [7,76]. Two chemical-based fingerprints were used in model development: MACCS molecular fingerprints and Morgan circular fingerprints. The server allows the user to select the preferred fingerprint, or both fingerprints, to be used in prediction generation. Results are shown as active or inactive for each individual CYP with assigned probabilities for the prediction. SuperCYPsPred is remarkably accurate; cross-validation accuracy averaged 0.930 (CYP3A4: 0.92; CYP2D6: 0.84; CYP2C19: 0.97; CYP2C9: 0.97; CYP1A2: 0.95) and external validation averaged 0.882 (CYP3A4: 0.86; CYP2D6: 0.80; CYP2C19: 0.95; CYP2C9: 0.90; CYP1A2: 0.90). Similar to DeepCYP, SuperCYPsPred is one of the most accurate free resources for CYP specificity, making it an excellent screening tool for pre-clinical drug design and development.

### 4.4. vNN-ADMET

vNN-ADMET is a free, publicly available web service (https://vnnadmet.bhsai.org/; accessed on 8 February 2021) that offers a wide range of ADMET predictions, including drug cytotoxicity, mutagenicity, cardiotoxicity, microsomal stability, hepatotoxicity, and cytochrome specificity [77]. Additionally, the webserver allows for public use of the versatile variable nearest neighbor (vNN) method for the custom design of novel models. The vNN method is similar to the k-nearest neighbor (kNN) method: a non-parametric method that uses pattern recognition of chemical features for classification and regression. One caveat of the kNN method is that it always gives a prediction for a test compound regardless of the degree of structural disagreement from the dataset. The vNN method is built using a predetermined similarity criterion, which only considers the nearest neighbors that share a defined degree of structural similarity (based on Tanimoto distance) to the input structure for prediction generation. A prediction is only made when valid nearest neighbors are identified. The Accelrys extended-connectivity fingerprints with a diameter of four chemical bonds (ECFP4) are used to identify structurally related compounds. Similar to the aforementioned machine learning models, a 10-fold cross-validation procedure was used to validate the model. The CYP inhibitor specificity results are portrayed as a categorical response (yes or no). vNN-ADMET is capable of making reliable CYP specificity predictions, with cross-validation accuracy averaging 0.890 (CYP3A4: 0.88; CYP2D6: 0.89; CYP2C19: 0.87; CYP2C9: 0.91; CYP1A2: 0.90), making the webserver an excellent resource for medicinal chemists.

### 4.5. AdmetSAR 2.0

AdmetSAR is a free resource useful in the prediction of ADMET properties of novel chemical entities (http://lmmd.ecust.edu.cn/admetsar2/; accessed on 8 February 2021) [78,79]. AdmetSAR 2.0 is an optimized version of AdmetSAR (1.0), now with 47 prediction models (formerly contained 27 models). Training data were procured from DrugBank, ChEMBL, CPDB, CYP450, and Tox21. Three chemical-based fingerprints were used in model development: MACCS molecular fingerprints, Morgan circular fingerprints, and AtomParis; all of which were implemented with RDKit. The scikit-learn package with python scripts was used to implement various machine learning algorithms, including random forests, support vector machines, and kNN. Supplementary details regarding method design and development are available online [78]. Six descriptors were used to define the applicability domain, including molecular weight, AlogP, number of atoms, number of rings, hydrogen bond acceptors, and hydrogen bond donors. The performance of binary models in CYP specificity prediction via AdmetSAR averaged 0.784 (CYP3A4: 0.645; CYP2D6: 0.855; CYP2C19: 0.806; CYP2C9: 0.802; CYP1A2: 0.815). Notably, CYP3A4 accuracy is lower than that of the previously mentioned platforms (0.645). One exciting novel feature of AdmetSAR 2.0 is ADMETopt—a module that allows users to perform molecule ADMET optimization by scaffold hopping. Scaffold hopping is a procedure that replaces the query molecule with a structurally similar scaffold that possess improved AMDET properties. AdmetSAR, and more specifically ADMETopt, is an excellent free resource that can be used in lead optimization.

### 4.6. SwissADME

SwissADME is a free, publicly available tool (http://www.swissadme.ch; accessed on 8 February 2021) capable of predicting physicochemical properties, pharmacokinetics, drug-likeness and medicinal chemistry friendliness [80]. Additionally, SwissADME provides graphical representations of biological barrier permeation (BOILED-Egg) and a Bioavailability Radar. Structures may be drawn directly into the webserver or submitted as a SMILES code, with categorical cytochrome inhibition predictions made on CYPs 3A4, 2D6, 2C19, 2C9, and 1A2. SwissADME was built using the PubChem Bioassay dataset, which contains 16,561 compounds with 50 chemical descriptors. SwissADME makes fast and reliable predictions, with an average 10-fold cross-validation accuracy of 0.794 (CYP3A4: 0.77; CYP2D6: 0.79; CYP2C19: 0.80; CYP2C9: 0.78; CYP1A2: 0.72); a 10-fold cross-validation AUROC (area under the receiver operating characteristic curve) average of 0.862 (CYP3A4: 0.85; CYP2D6: 0.85; CYP2C19: 0.86; CYP2C9: 0.85; CYP1A2: 0.90); an external validation accuracy average of 0.788 (CYP3A4: 0.78; CYP2D6: 0.81; CYP2C19: 0.80; CYP2C9: 0.71; CYP1A2: 0.84), and an external AUROC average of 0.864 (CYP3A4: 0.86; CYP2D6: 0.87; CYP2C19: 0.87; CYP2C9: 0.81; CYP1A2: 0.91). SwissADME affords unique advantages, allowing for the input of multiple molecules and for the user to export data in various file formats. SwissADME analyzes small molecule composition to determine whether it violates or is in compliance with Lipinski’s Rule of Five and PAINS, two drug triage tools used in industry to predict drug likeness.

### 4.7. CypRules

CypRules is a freely available webserver (http://cyprules.cmdm.tw/; accessed on 8 February 2021) that predicts inhibitors and non-inhibitors of the five major CYPs 3A4, 2D6, 2C19, 2C9, and 1A2 [81]. CypRules utilizes the same dataset as SwissADME for predictions; however, CypRules utilizes decision trees in combination with the theory of information entropy [13,81]. CypRules uses three descriptor tools: PubChem 2D Fingerprint, PaDEL-Descriptor, and Mold2 [75,82]. Classification models utilize a C5.0 algorithm, which chooses an attribute from the data that most effectively splits the initial dataset into enriched subsets [81]. CypRules makes accurate predictions, with an average accuracy of 0.812 (CYP3A4: 0.73; CYP2D6: 0.90; CYP2C19: 0.86; CYP2C9: 0.77; CYP1A2: 0.80). An added benefit of CypRules is the ability to perform virtual high-throughput screening of a large set of testing compounds.

### 4.8. CypReact

CypReact is a freely available tool (https://bitbucket.org/Leon_Ti/cypreact; accessed on 8 February 2021) capable of predicting reactants of the nine most important CYP enzymes: CYPs 3A4, 2E1, 2D6, 2C19, 2C9, 2C8, 2B6, 2A6, and 1A2 [83]. In addition to predicting whether a test item will serve as a reactant of a given cytochrome, CypReact is also capable of predicting sites of metabolism (SoMs), as well as the end result of the interaction (“endpoint prediction”). This allows for the user to generate predictions on the query molecule, as well as prospective metabolites. CypReact generates binary data on all parameters (1 = true; 0 = false). The CypReact dataset was built using 1632 compounds extracted from Zaretzki et al. [84]. An additional 1053 non-reactant compounds were gathered in order to improve the quality and predictive capacity of the dataset. Three chemical-based fingerprints were used in model development: MACCS molecular fingerprints, PubChem fingerprints, and ClassyFire27 fingerprint. CypReact employs an LBM learning algorithm that considers a set of 2279 features of dataset compounds, including 36 physiochemical properties. Additional information on model training and dataset selection is available online [83]. CypReact makes reliable predictions, with AUROC values ranging from 0.83 to 0.92. Furthermore, CypReact outperforms other reactant prediction tools such as ADMET Predictor (AUROC = 0.75) and SMARTCyp (AUROC = 0.53). ADMET Predictor and SMARTCyp are reviewed elsewhere [13,85,86]. The resulting metabolites may be screened for CYP inhibition specificity and toxicity risk using the previously described platforms. One criticism of CypReact is the user interface. The platform utilizes bitbucket, requiring the use of rudimentary coding skills, making the model less user-friendly than the previously described methods. However, the inclusion of SoM prediction(s) makes CypReact a gold standard prediction model.

## 5. Conclusions

More than half of drug failures are a result of poor ADMET. More specifically, drug–drug interactions as a result of major cytochrome inhibition are commonplace and can lead to life-threatening adverse drug reactions. A majority of pre-clinical-stage drug discovery programs focus solely on binding affinity and selectivity [50]. Although binding affinity is of utmost importance, ADMET properties ensure that a drug is properly absorbed, distributed, and cleared; features that equally contribute to the success of a drug in the clinic. As such, in silico approaches have been developed to be introduced during early drug screening, with the goal of reducing the rate of drug attrition (Table 2). 

In silico approaches utilize a wide range of molecular descriptors to build and train the models. Physiochemical descriptors are considered to be highly predictive of CYP specificity and binding affinity. Thus, we explored the ChEMBL database, which contains over 10,000 CYP inhibitors with individual items of data on molecular weight, lipophilicity (ALogP), and half-maximal inhibitory concentration (IC_50_). Although CYP inhibitors share common physiochemical features, molecular weight and lipophilicity alone are unable to predict CYP half-maximal inhibitory concentration. CYP3A4 and CYP2C9 inhibitors demonstrated a weak statistically significant negative relationship between molecule size, lipophilicity, and IC50 (i.e., larger, more lipophilic molecules tend to have lower IC50 values); however, the exact opposite trend was observed for CYP1A2. No relationship was identified for CYPs 2D6 and 2C19. We then explored the common structural features of major cytochrome inhibitors, which are summarized above (Table 1). These features are critically important in making predictions; however, these data alone have limited predictive value without the help of machine learning-based methods. In this review, we highlight eight machine learning-based approaches for the prediction of cytochrome inhibition. These eight platforms are publicly available and free to the user. Based on the average accuracies provided in the literature, SuperCYPsPred (0.930) appears to be the most accurate tool for predicting CYP inhibition, followed by vNN-ADMET (0.890) and DeepCYP (0.864). It should be noted, however, that each of the mentioned platforms are capable of making reliable predictions on CYP inhibitor specificity and possess their own unique features. pkCSM and AdmetSAR 2.0 are capable of predicting a suite of ADMET properties. More specifically, pkCSM is capable of predicting the outcomes of commonly used in vitro ADMET assays, as well as human gastrointestinal absorption and toxicity. Additionally, pkCSM provides the data on human volume of distribution and clearance, which can be used to estimate drug half-life. Although pkCSM may not be the most accurate machine learning-based platform discussed in this review, it is capable of making rapid predictions on a broad range of parameters and is user-friendly. In addition to predicting ADMET parameters, AdmetSar 2.0 hosts ADMETopt, a platform that assists the user with lead optimization. All of the platforms described assign binary “yes” or “no” predictions for CYP inhibition, with the exception of DeepCyp and SuperCypsPred; these tools assign probabilities to their predictions. Probability assignment is useful, as it allows the user to gauge the relative accuracy and utility of the prediction. SwissADME makes predictions on physicochemical properties, pharmacokinetics, and drug likeness. Physiochemical properties such as solubility are important for drug formulation and in vivo exposure. Additionally, SwissADME assesses whether a test molecule violates industry standard rules such as Lipinski’s Rule of Five. These predictions are important for predicting the future success of drug candidates and should be considered during the early stages of drug development. SwissADME and CypRules are capable of performing high-throughput screening of large drug libraries. Lastly, CypReact features site of metabolism and metabolite predictions. These predictions can be used to assess CYP reactivity and prospective metabolite-driven toxicities. The goal of this article is to promote the use of ADMET prediction software early in the drug design process in order to improve drug discovery outcomes.

## Figures and Tables

**Table 1:** Key characteristics of major cytochrome p450 enzymes and their inhibitors.

	Enzyme		Inhibitors	
CYP	Cavity Size (Å^3^)	Description	Description	Common Descriptors
3A4	1173–2862	Large, open, flexible, aromatic, diverse range of substrates, oxidation site is commonly a nitrogen or allylic position	Large, structurally diverse, lipophilic, aromatic, highly flexible, high hydrogen bond accepting capacity	One of more aromatic moieties, furan rings, tertiary amines, acetylene groups
2D6	510	Flat, restricted volume, acidic, aromatic, site of oxidation proximal to a primary or secondary amine	Flat, planar, aromatic structures capable of procuring a positive charge, with 2–3 hydrogen bond acceptors	One of more aromatic moieties, heterocycles, primary or secondary amines capable of carrying a positive charge
2C19	Not reported	Aromatic, moderately flexible, similar to CYP2C9	Medium sized molecules, variable lipophilicity, aromatic, oxidation site close to two hydrogen bond acceptors	Several aromatic moieties, heterocycles, carbonyl groups, and aromatic nitrogen atoms
2C9	978–1271	Larger cavity volume, moderately flexible	Aromatic, lipophilic, moderately flexible, several hydrogen bond acceptors	Aromatic, heterocycles, aromatic nitrogens, primary amines, and halogens
1A2	375–390	Small cavity volume, planar, rigid	Small, planar, aromatic, lipophilic, slightly acidic	Several aromatic moieties, heterocycles, secondary amines, and halogens

**Table 2:** Summary of publicly available machine learning-based methods for cytochrome inhibition and metabolism.

Name	CYPs	Prediction Type	ML Method	No. Structures	Avg. Accuracy ^1^	Additional Features
pkCSM	3A4, 2D6, 2C19, 2C9, 1A2	Inhibition	Graph-based signatures	18,000	0.810 (0.780–0.853)	Comprehensive ADMET predictions (23 total)
DeepCYP	3A4, 2D6, 2C19, 2C9, 1A2	Inhibition	Multitask autoencoder deep neural network	13,000	0.864 (0.809–0.968)	Assigns probabilities for CYP inhibition
SuperCYPs-Pred	3A4, 2D6, 2C19, 2C9, 1A2	Inhibition	Random forests	41,963 ^2^	0.930 (0.840–0.970)	Assigns probabilities for CYP inhibition
vNN-ADMET	3A4, 2D6, 2C19, 2C9, 1A2	Inhibition	Variable nearest neighbors	6261	0.890 (0.870–0.910)	
AdmetSAR 2.0	3A4, 2D6, 2C19, 2C9, 1A2	Inhibition	Random forests, support vector machines, k-nearest neighbors	96,000 ^3^	0.784 (0.645–0.855)	Comprehensive ADMET predictions (47 total); ADMETopt for lead optimization
SwissADME	3A4, 2D6l 2C19, 2C9, 1A2	Inhibition	Support vector machines	16,561 ^4^	0.794 (0.720–0.800)	Predictions of physicochemical properties, pharmacokinetics, and drug likeness; high throughput
CypRules	3A4, 2D6, 2C19, 2C9, 1A2	Inhibition	Decision trees	16,561	0.812 (0.730–0.900)	High throughput
CypReact	3A4, 2E1, 2D6,2C19, 2C9, 2C8, 2B6, 2A6, 1A2	Sites of metabolism	LBM learning algorithm	2685	Unavailable	Metabolite predictions; Additional CYPs

^1^ Cross-validation accuracy. ^2^ Sum of the number of compounds used for all five major cytochromes. The dataset includes inhibitors and non-inhibitors and may include duplicates. ^3^ Includes compounds used to build ADMET models in addition to CYP inhibition models. ^4^ Includes compounds used to establish physicochemical properties, pharmacokinetics, drug likeness and medicinal chemistry friendliness models.

## Data Availability

Individual data are available via FigShare [87].

## Supplementary Materials

The following are available online at https://www.mdpi.com/article/10.3390/ph14050472/s1, Table S1: CYP3A4 Multiple Linear Regression; Table S2: CYP2D6 Multiple Linear Regression; Table S3: CYP2C19 Multiple Linear Regression; Table S4: CYP2C9 Multiple Linear Regression; Table S5: CYP3A4 Multiple Linear Regression.
