# Supplementary material for: Descriptors of Cytochrome Inhibitors and Useful Machine Learning Based Methods for the Design of Safer Drugs
Source: Pharmaceuticals (Basel). 2021 May 17;14(5):472. doi: 10.3390/ph14050472 (PMC8156202; doi:10.3390/ph14050472)
Supplement: Supplementary file 1 [file pharmaceuticals-14-00472-s001.zip › S1 (CYP3A4).pdf]

CYP3A4 Multiple Linear Regression

Variables Entered/Removed[a]

Model	Variables Entered	Variables Removed	Method
1	AlogP, MW[b]	.	Enter

a. Dependent Variable: IC50
b. All requested variables entered.

Model Summary[b]

Model	R	R Square	Adjusted R Square	Std. Error of the Estimate	Durbin-Watson
1	.136[a]	.019	.018	81771.77535	1.942

a. Predictors: (Constant), AlogP, MW
b. Dependent Variable: IC50

ANOVA[a]

Model		Sum of Squares	df	Mean Square	F	Sig.
1	Regression	485068852346.895	2	242534426173.447	36.272	.000[b]
	Residual	25623140270509.418	3832	6686623243.870		
	Total	26108209122856.312	3834			

a. Dependent Variable: IC50
b. Predictors: (Constant), AlogP, MW

Coefficients[a]

Model	Unstandardized Coefficients		Standardized Coefficients	t	Sig.	95.0% Confidence Interval for B	
	B	Std. Error	Beta			Lower Bound	Upper Bound

1	(Constant)	59368.675	5542.188		10.712	.000	48502.753	70234.596
	MW	-54.874	10.970	-.087	-5.002	.000	-76.382	-33.366
	AlogP	-4590.356	1060.100	-.076	-4.330	.000	-6668.770	-2511.943

a. Dependent Variable: IC50

Residuals Statistics[a]

	Minimum	Maximum	Mean	Std. Deviation	N
Predicted Value	-29579.5566	52935.0313	13648.4853	11248.00860	3835
Residual	-50869.33594	3949929.50000	.00000	81750.44451	3835
Std. Predicted Value	-3.843	3.493	.000	1.000	3835
Std. Residual	-.622	48.304	.000	1.000	3835

a. Dependent Variable: IC50

Notes

Output Created		03-MAY-2021 21:08:12
Comments		
Input	Active Dataset	DataSet0
	Filter	<none>
	Weight	<none>
	Split File	<none>
	N of Rows in Working Data File	3839
Missing Value Handling	Definition of Missing	User-defined missing values are treated as missing.
	Cases Used	Statistics are based on cases with no missing values for any variable used.
Syntax		REGRESSION<br>/MISSING LISTWISE<br>/STATISTICS COEFF OUTS CI(95) R ANOVA<br>/CRITERIA=PIN(.05) POUT(.10)<br>/NOORIGIN<br>/DEPENDENT IC50<br>/METHOD=ENTER MW AlogP<br>/RESIDUALS DURBIN.
Resources	Processor Time	00:00:00.04
	Elapsed Time	00:00:00.00
	Memory Required	2912 bytes
	Additional Memory Required for Residual Plots	0 bytes
