# Supplementary material for: Descriptors of Cytochrome Inhibitors and Useful Machine Learning Based Methods for the Design of Safer Drugs
Source: Pharmaceuticals (Basel). 2021 May 17;14(5):472. doi: 10.3390/ph14050472 (PMC8156202; doi:10.3390/ph14050472)
Supplement: Supplementary file 1 [file pharmaceuticals-14-00472-s001.zip › S2 (CYP2D6).pdf]

CYP2D6 Multiple Linear Regression

Variables Entered/Removed[a]

Model	Variables Entered	Variables Removed	Method
1	ALogP, MW[b]	.	Enter

a. Dependent Variable: IC50
b. All requested variables entered.

Model Summary[b]

Model	R	R Square	Adjusted R Square	Std. Error of the Estimate	Durbin-Watson
1	.024[a]	.001	.000	17591.99529	.002

a. Predictors: (Constant), ALogP, MW
b. Dependent Variable: IC50

ANOVA[a]

Model		Sum of Squares	df	Mean Square	F	Sig.
1	Regression	415446015.685	2	207723007.842	.671	.511[b]
	Residual	691993475093.035	2236	309478298.342		
	Total	692408921108.720	2238			

a. Dependent Variable: IC50
b. Predictors: (Constant), ALogP, MW

Coefficients[a]

Model		Unstandardized Coefficients		Standardized Coefficients	t	Sig.	95.0% Confidence Interval for B	
		B	Std. Error	Beta			Lower Bound	Upper Bound
1	(Constant)	11752.983	1577.178		7.452	.000	8660.096	14845.869

MW	3.781	4.036	.023	.937	.349	-4.135	11.696
ALogP	-356.536	340.955	-.025	-1.046	.296	-1025.157	312.085

a. Dependent Variable: IC50

Residuals Statistics[a]

	Minimum	Maximum	Mean	Std. Deviation	N
Predicted Value	10357.5625	13680.0479	11889.1630	430.85115	2239
Residual	-13562.78906	88856.10156	.00000	17584.13295	2239
Std. Predicted Value	-3.555	4.157	.000	1.000	2239
Std. Residual	-.771	5.051	.000	1.000	2239

a. Dependent Variable: IC50
