# Supplementary material for: Descriptors of Cytochrome Inhibitors and Useful Machine Learning Based Methods for the Design of Safer Drugs
Source: Pharmaceuticals (Basel). 2021 May 17;14(5):472. doi: 10.3390/ph14050472 (PMC8156202; doi:10.3390/ph14050472)
Supplement: Supplementary file 1 [file pharmaceuticals-14-00472-s001.zip › S3 (CYP2C19).pdf]

CYP2C19 Multiple Linear Regression

Variables Entered/Removed[a]

Model	Variables Entered	Variables Removed	Method
1	ALogP, MW[b]	.	Enter

a. Dependent Variable: IC50
b. All requested variables entered.

Model Summary[b]

Model	R	R Square	Adjusted R Square	Std. Error of the Estimate	Durbin-Watson
1	.125[a]	.016	.014	17011.59560	.029

a. Predictors: (Constant), ALogP, MW
b. Dependent Variable: IC50

ANOVA[a]

Model		Sum of Squares	df	Mean Square	F	Sig.
1	Regression	5059853107.380	2	2529926553.690	8.742	.000[b]
	Residual	318912612106.991	1102	289394384.852		
	Total	323972465214.371	1104			

a. Dependent Variable: IC50
b. Predictors: (Constant), ALogP, MW

Coefficients[a]

Model		Unstandardized Coefficients		Standardized Coefficients			95.0% Confidence Interval for B	
		B	Std. Error	Beta	t	Sig.	Lower Bound	Upper Bound
1	(Constant)	16529.941	2216.776		7.457	.000	12180.363	20879.519

MW	12.556	5.850	.075	2.146	.032	1.078	24.033
ALogP	-1955.249	467.643	-.147	-4.181	.000	-2872.821	-1037.677

a. Dependent Variable: IC50

Residuals Statistics[a]

	Minimum	Maximum	Mean	Std. Deviation	N
Predicted Value	7993.3345	26744.5000	13416.2302	2140.84102	1105
Residual	-18089.91406	85361.92969	.00000	16996.17956	1105
Std. Predicted Value	-2.533	6.226	.000	1.000	1105
Std. Residual	-1.063	5.018	.000	.999	1105

a. Dependent Variable: IC50
