# Supplementary material for: Descriptors of Cytochrome Inhibitors and Useful Machine Learning Based Methods for the Design of Safer Drugs
Source: Pharmaceuticals (Basel). 2021 May 17;14(5):472. doi: 10.3390/ph14050472 (PMC8156202; doi:10.3390/ph14050472)
Supplement: Supplementary file 1 [file pharmaceuticals-14-00472-s001.zip › S4 (CYP2C9).pdf]

CYP2C9 Multiple Linear Regression

Variables Entered/Removed[a]

Model	Variables Entered	Variables Removed	Method
1	ALogP, MW[b]	.	Enter

a. Dependent Variable: IC50
b. All requested variables entered.

Model Summary[b]

Model	R	R Square	Adjusted R Square	Std. Error of the Estimate	Durbin-Watson
1	.132[a]	.018	.017	16089.10739	.033

a. Predictors: (Constant), ALogP, MW
b. Dependent Variable: IC50

ANOVA[a]

Model		Sum of Squares	df	Mean Square	F	Sig.
1	Regression	9707574923.667	2	4853787461.834	18.751	.000[b]
	Residual	543345831594.114	2099	258859376.653		
	Total	553053406517.782	2101			

a. Dependent Variable: IC50
b. Predictors: (Constant), ALogP, MW

Coefficients[a]

Model		Unstandardized Coefficients		Standardized Coefficients	t	Sig.	95.0% Confidence Interval for B	
		B	Std. Error	Beta			Lower Bound	Upper Bound
1	(Constant)	21177.983	1597.119		13.260	.000	18045.882	24310.085

MW	-12.643	4.029	-.079	-3.138	.002	-20.544	-4.743
ALogP	-883.258	300.396	-.074	-2.940	.003	-1472.363	-294.154

a. Dependent Variable: IC50

Residuals Statistics[a]

	Minimum	Maximum	Mean	Std. Deviation	N
Predicted Value	2146.8320	19349.5176	11908.4304	2149.52425	2102
Residual	-15888.91211	94171.64063	.00000	16081.44774	2102
Std. Predicted Value	-4.541	3.462	.000	1.000	2102
Std. Residual	-.988	5.853	.000	1.000	2102

a. Dependent Variable: IC50
