# Supplementary material for: Descriptors of Cytochrome Inhibitors and Useful Machine Learning Based Methods for the Design of Safer Drugs
Source: Pharmaceuticals (Basel). 2021 May 17;14(5):472. doi: 10.3390/ph14050472 (PMC8156202; doi:10.3390/ph14050472)
Supplement: Supplementary file 1 [file pharmaceuticals-14-00472-s001.zip › S5 (CYP1A2).pdf]

CYP1A2 Multiple Linear Regression

Variables Entered/Removed[a]

Model	Variables Entered	Variables Removed	Method
1	ALogP, MW[b]	.	Enter

a. Dependent Variable: IC50
b. All requested variables entered.

Model Summary[b]

Model	R	R Square	Adjusted R Square	Std. Error of the Estimate	Durbin-Watson
1	.309[a]	.095	.094	17520.93715	.167

a. Predictors: (Constant), ALogP, MW
b. Dependent Variable: IC50

ANOVA[a]

Model		Sum of Squares	df	Mean Square	F	Sig.
1	Regression	33624835233.625	2	16812417616.812	54.767	.000[b]
	Residual	319262568303.933	1040	306983238.754		
	Total	352887403537.557	1042			

a. Dependent Variable: IC50
b. Predictors: (Constant), ALogP, MW

Coefficients[a]

Model		Unstandardized Coefficients		Standardized Coefficients	t	Sig.	95.0% Confidence Interval for B	
		B	Std. Error	Beta			Lower Bound	Upper Bound
1	(Constant)	-8252.088	2259.915		-3.652	.000	-12686.601	-3817.576

	MW	70.048	7.039	.338	9.951	.000	56.235	83.861
	ALogP	-1055.146	496.213	-.072	-2.126	.034	-2028.838	-81.454

a. Dependent Variable: IC50

Residuals Statistics[a]

	Minimum	Maximum	Mean	Std. Deviation	N
Predicted Value	-2393.7456	37207.6719	11717.3731	5680.62634	1043
Residual	-34362.86719	86729.93750	.00000	17504.11436	1043
Std. Predicted Value	-2.484	4.487	.000	1.000	1043
Std. Residual	-1.961	4.950	.000	.999	1043

a. Dependent Variable: IC50
